# Determination of oligomerization state of Drp1 protein in living cells at nanomolar concentrations

**DOI:** 10.1038/s41598-019-42418-0

**Published:** 2019-04-11

**Authors:** Karina Kwapiszewska, Tomasz Kalwarczyk, Bernadeta Michalska, Krzysztof Szczepański, Jędrzej Szymański, Paulina Patalas-Krawczyk, Tomasz Andryszewski, Michalina Iwan, Jerzy Duszyński, Robert Hołyst

**Affiliations:** 1 Institute of Physical Chemistry, Polish Academy of Sciences, Kasprzaka 44/52, 01-224 Warsaw, Poland; 2 Nencki Institute of Experimental Biology, 3 Pasteur St, 02-093 Warsaw, Poland

## Abstract

Biochemistry in living cells is an emerging field of science. Current quantitative bioassays are performed *ex vivo*, thus equilibrium constants and reaction rates of reactions occurring in human cells are still unknown. To address this issue, we present a non-invasive method to quantitatively characterize interactions (equilibrium constants, K_D_) directly within the cytosol of living cells. We reveal that cytosolic hydrodynamic drag depends exponentially on a probe’s size, and provide a model for its determination for different protein sizes (1–70 nm). We analysed oligomerization of dynamin-related protein 1 (Drp1, wild type and mutants: K668E, G363D, C505A) in HeLa cells. We detected the coexistence of wt-Drp1 dimers and tetramers in cytosol, and determined that K_D_ for tetramers was 0.7 ± 0.5 μM. Drp1 kinetics was modelled by independent simulations, giving computational results which matched experimental data. This robust method can be applied to *in vivo* determination of K_D_ for other protein-protein complexes, or drug-target interactions.

## Introduction

Biomolecular interactions are the basic components of life processes^1^. Cellular metabolism, function, division, and fate rely on a network of interconnected biochemical reactions, and any disruption of this fragile balance can lead to pathological changes in the cell or a whole organism. For example, the latest research into the kinesin-1 molecular motor reveals that only a 2-fold increase in cytoplasmic viscosity can stop motion of motor proteins^2^. Efforts to both detect these interactions and quantify their affinities and reaction rates are crucial to understanding metabolism and its related diseases. Many advanced methods have been developed to identify and quantify biomolecular interactions in living cells^3,4^, but most approaches (e.g. mass spectrometry, yeast two-hybrid system, confocal imaging) concerning *in situ* interactions are qualitative or semi-quantitative^5^. Fully quantitative assays such as surface plasmon resonance and biochemical tests require purified and processed samples (by fixation or extraction of the material)^4^, which prohibits their application to living cells. These limitations have inhibited characterization of *in vivo* interactions’ quantitative dynamics as measured by their equilibrium constants and reaction rates^6^; there are few reports on the quantification of biochemical reaction kinetics directly within living human cells^7–9^. Förster Resonance Energy Transfer (FRET) is the leading technique used for this purpose^10^: in principle, each of the interacting molecules is labelled with one dye of a FRET pair. In the *in vivo* experiments^7^, one of the molecules is initially expressed or introduced into the cells and the other added during the experiment. The FRET signal’s presence and change over time can be interpreted in terms of reaction kinetics^7,9^. This technique has so far been applied to the binding of bacterial proteins^7^, conformational changes of a human protein^8^ in HeLa cells, and the interaction between an enzyme and a product in HEK293T cells^9^. However, these crucial works are still isolated, most probably because of the laborious protocols. FRET experiments require separate labelling of two different probes, and simultaneous probe introduction and measurement can be troublesome due to technical issues. Thus, experiments on protein dynamics in living human cells, though crucial, are still difficult to conduct.

As a complement to FRET, we propose fluorescence correlation spectroscopy (FCS) applied to the quantification of reaction kinetics directly in the cytoplasm of living cells. FCS enables the determination of a fluorescent probe’s diffusion coefficient directly in living cells^11–14^. FCS is non-invasive, applicable to low, physiologically relevant concentrations of probes (1–100 nM), and requires relatively mild experimental conditions (low laser power, short acquisition times)^15,16^. Previous use of FCS in living cells has been limited to reporting diffusion coefficients of probes^17^, rather than more in-depth analysis on the probes’ conformation or binding. The limitation lies in understanding cytoplasmic hydrodynamic drag, f = 6πη_eff_r_p_, where r_p_ is hydrodynamic radius of a probe and η_eff_ is viscosity of the cytoplasm. According to the Stokes-Sutherland-Einstein relation^18,19^, the diffusion coefficient depends on hydrodynamic drag. Since η_eff_ of the cellular interior is unknown, probe size cannot be determined either. Our previous research indicates that apart from clear spatial heterogeneity (according to compartmentalization)^20^, structural heterogeneity also affects η_eff_ of cytoplasm^21,22^. According to our findings, cytosolic η_eff_ exponentially depends on probe size and can be described using Eq. 1:

1 $${\eta }_{eff}={\eta }_{0}\,A\,exp[{(\frac{{\xi }^{2}}{{{R}_{h}}^{2}}+\frac{{\xi }^{2}}{{{r}_{p}}^{2}})}^{-a/2}]$$

where η_0_ is the viscosity of a reference buffer, A is a preexponential factor of the order of one, ξ and R_h_ are length scales characteristic for a given system, and a is an exponent smaller than 1. This model includes all factors acting on a neutral probe, *i*.*a*. macromolecular crowding^22^. All parameters of Eq. 1 depend on cell type and cell culture conditions^21^; every experiment, in which cytoplasmic viscosity is a parameter, should be preceded with careful determination of η_eff_ at the length scale of interest.

**Figure 1:**
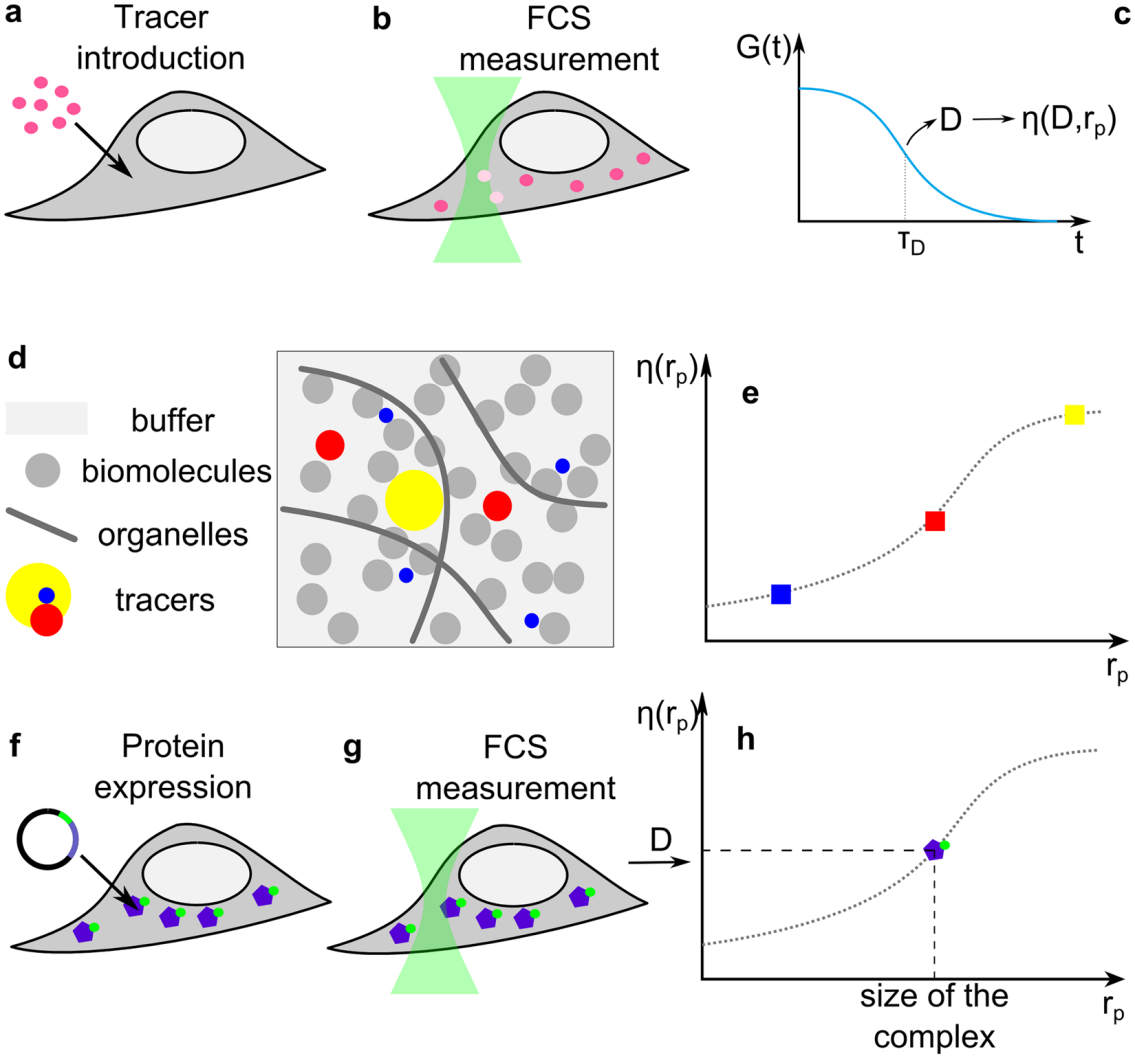
Principle of FCS-based determination of protein dynamics in cytoplasm of a living cell. (**a**) Fluorescent tracers of known hydrodynamic radii (r_p_) are introduced into cytosol and (**b**) FCS signal is measured. (**c**) Diffusion coefficient is obtained from FCS and η_eff_ can be calculated for the given r_p_. (**d**) Cytoplasm is a complex environment, where tracers of different sizes experience various drag. Several tracers should be measured separately to obtain (**e**) η_eff_(r_p_) dependence for the particular cell type according to Eq. 1. Next, (**f**) protein of interest with fluorescent tag is expressed in the cell and (**g**) FCS measurements are conducted. (**h**) Diffusion coefficients obtained from data fitting can be interpreted using (r_p_) dependence, and thus protein complex size can be calculated.

In this paper we show that determination of diffusion coefficients of a protein and its complexes can be used to quantify these protein interactions directly in living cells. This principle is presented briefly in Fig. 1. We chose the oligomerization of dynamin-related protein 1 (Drp1) as a process of interest^23^. Drp1 is involved in fission of mitochondria^24,25^, the dynamics of which are important for many cellular processes (cellular energetics, intrinsic apoptosis etc.)^23–26^. It is known that Drp1 forms dimers and tetramers that assemble on membranes^23^, but it remains to be seen whether tetramers occur in cytoplasm or are formed directly on membranes^26,27^. The kinetic details of Drp1 oligomerization are also unknown.

We used tracers of known hydrodynamic radii to determine η_eff_ of HeLa cells’ cytosol at the length scales expected for monomer and oligomers of Drp1 (1–10 nm). Parameters of Eq. 1 were then fitted to these experimental data and expected diffusion coefficients for Drp1 oligomers were calculated. EGFP-tagged Drp1 mutants (K668E, G363D and C505A) were expressed in HeLa cells to validate FCS protocols. Finally, the EGFP-Drp1 wild type was evaluated with the same protocol and the equilibrium between dimer and tetramer was found in cytosol of HeLa. FCS data allowed determination of each oligomer’s concentration, and thus K_D_ (dissociation constant) values.

## Results

### The hydrodynamic drag of cytoplasm in HeLa cells depends on length scale

**Figure 2:**
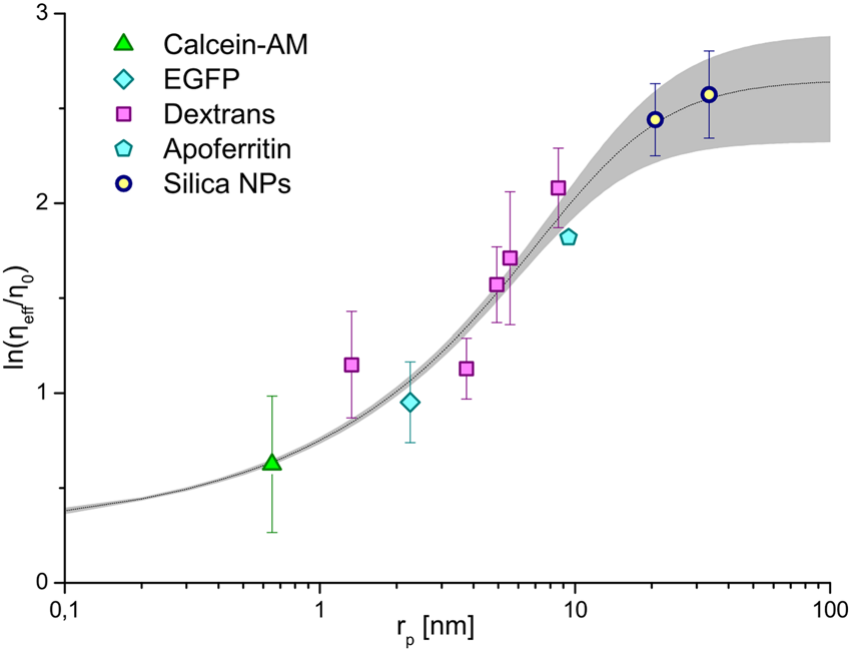
Length-scale dependent η_eff_ measured in cytosol of HeLa cells in 36 °C. Experimental points (see Table SI2.1) are plotted as scatter, error bars correspond to standard deviations (N > 15). Fit of length-scale dependent hydrodynamic drag model (Eq. 1) is presented as a dotted line with shade representing error (calculated using total differential method). η_eff_ increases from approx. 1.9 viscosities of water for calcein (r_p_ = 0.65 nm), to approx. 12 viscosities of water for nanoparticles (r_p_ > 20 nm).

Tracers used for probing *η*_*eff*_ were chosen according to their expected inertness in the cytosol. Hydrodynamic tracer characterizations are detailed in Supplementary Information (SI, section SI1)^28^. Each tracer was measured by FCS in cytosol of HeLa cells (see SI2 for details). *η*_*eff*_ was calculated for each length scale (*r*_*p*_) and results were plotted in Fig. 2. It is clear that the larger a probe that is introduced into the cytoplasm, the greater drag it experiences. These results reflect the model (Fig. 1d), in which small molecules move freely between macromolecules, but larger probes experience macromolecular crowding and thus increasing resistance to mobility. The length-scale dependent hydrodynamic drag model (Eq. 1) was fitted to experimental data from HeLa cells with following fit parameters: *A* = 1.3 ± 0.3, *ξ* = 3.16 ± 0.14 nm, R_h_ = 12.9 ± 2.3 nm, and a = 0.62 ± 0.07. This proposed model matches experimental data well, as displayed in Fig. 2. One should be aware that these parameters fit the case of HeLa cells in certain culture conditions, and can be used for further experiments in HeLa cells only. For any other cell line, temperature, or other differing conditions, *η*_eff_ should be determined separately.

### Expected diffusion coefficients of Drp1 oligomers in cytoplasm of HeLa cells

**Figure 3:**
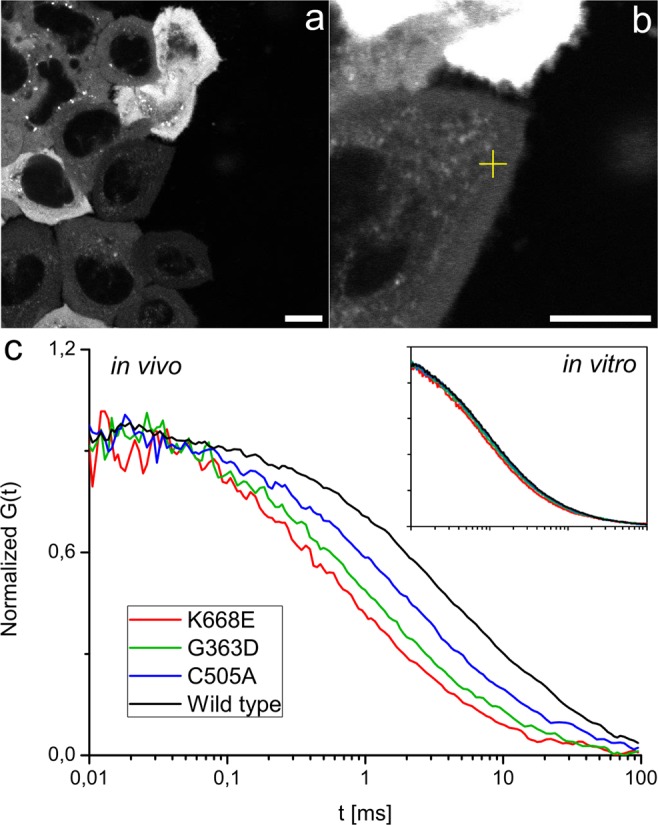
FCS measurements in HeLa cells. (**a**) Confocal image of EGFP-Drp1 expressing cells, variety of expression levels can be noticed. FCS measurements were performed in cells with low expression, which provided approx. 10 fluorescent molecules in focal volume. (**b**) Close-up with a measurement spot marked with a cross. Scale bars correspond to 10 μm. (**c**) Example autocorrelation curves obtained for different mutants of Drp1. Each of the variants can be distinguished: K668E (monomer), G363D (dimer), C505A (dimer with higher membrane affinity) and wild type (tetrameric form and highest membrane affinity expected). All curves obtained with the same calibration settings. Insert presents autocorrelation curves obtained for cell extracts of the same mutants in – lack of the complex cytoplasmic architecture disables possibility of distinction of oligomers.

According to existing knowledge, Drp1 can occur in a cytoplasm as a monomer, dimer or tetramer^26^. r_p_ of the monomer, dimer and tetramer of EGFP tagged Drp1 were calculated using HydroPro software (see SI3). Expected diffusion coefficients were calculated according to Eq. 1, with parameters derived for HeLa cells (D_HeLa_, see Table 1). Table 1 also contains diffusion coefficients calculated with the assumption of constant cytosolic viscosity (D_x_). Because more accurate data is unavailable, the assumption of a constant viscosity of cytosol, usually probed by EGFP protein, is a common practice in biophysical studies^29,30^. Imprecise quantification of cytosolic *η*_eff_ for a given length scale can lead to a serious miscalculation of an expected diffusion coefficient (reaching nearly 300% for the tetramer, shown in Table 1). An additional outcome of the length-scale dependency of cytosolic *η*_eff_ are differences between diffusion coefficients of subsequent oligomers. If *η*_eff_ were constant, the difference between diffusion coefficients of monomer and dimer would be 26%, and 24% between dimer and tetramer (see Table 1). Such differences would be indistinguishable in FCS or analogous techniques, but according to Eq. 1 the actual differences are 40% and 37% respectively. Thus, diffusion times of these oligomers are most likely to be distinguished. Figure 3c presents that cytoplasmic structure promotes FCS-based distinction of Drp1 oligomers. FCS performed in lysates of cells expressing EGFP-tagged Drp1 mutants (K668E, G363D, C505A and wild type) revealed hardly any difference between variants. On the other hand, FCS curves acquired in viable cells differed distinctly, which enabled further analysis.

**Table 1:** Hydrodynamic characterization of oligomers of Drp1 protein.

Drp1	r_p_ [nm]	D_HeLa_ [μm^2^/s]	D_x_ [μm^2^/s]
Monomer	4.74	15.1 ± 0.5	28.9
Dimer	6.41	9.1 ± 0.5	21.4
Tetramer	8.43	5.7 ± 0.4	16.3

D_HeLa_ - diffusion coefficients calculated according to Eqn. 1 with parameters obtained for HeLa cytosol in 36 °C (Fig. 2).

D_x_ - diffusion coefficients calculated assuming constant viscosity of cytosol. Viscosity experienced by EGFP protein was 2.340.

### K668E mutation of Drp1 prevents its oligomerization

According to literature^31^, it is expected that K668E-Drp1 would remain monomeric. FCS autocorrelation curves of EGFP-K668E-Drp1 in cytosol were fitted using a two-component free diffusion model (see SI4). A summary of the results is presented in Table 2. The diffusion coefficient is in the range of 15.54 ± 0.13 μm^2^/s, which suits the expected value (15.1 ± 0.5, Table 1). These results indicate that η_eff_ of cytosol of HeLa cells at the length scale of Drp1 monomer was predicted correctly. FCS data indicate the existence of a second, slow diffusing component (diffusion time of milliseconds, see SI4). The most probable explanation for the existence of this component is nonspecific binding of Drp1 with cytoplasmic content (membranes or proteins). Highly variable, long diffusion times and a relatively high fraction (approx. 80%) of the free diffusing monomer can be attributed to non-specificity of these binding events.

**Table 2:** Summary of Drp1 measurements in HeLa cells.

Drp1 mutant	D_FCS_ [μm^2^/s]	Amplitude*	Phenotype
K668E	15.54 ± 0.13	0.80 ± 0.09	monomer, low affinity to membranes
G363D	9.14 ± 0.18	0.67 ± 0.10	dimer, low affinity to membranes
C505A	8.85 ± 0.12	0.44 ± 0.12	dimer, higher affinity to membranes
Wild type	9.1 ± 0.50	0.34 ± 0.09	dimer and tetramer, high affinity to membranes
	5.7 ± 0.40	0.15 ± 0.05	

D_FCS_ - diffusion coefficients calculated from autocorrelation curves with the respect to the calibration data (see SI1).

*Amplitude is proportional to relative concentration of components.

### G363D and C505A mutations of Drp1 enable dimerization

G363D-Drp1 mutant is reported to be a dimer with no mitochondrial membrane affinity^32,33^. FCS autocorrelation curves recorded for EGFP-G363D-Drp1 in HeLa cells were fitted using a two component free diffusion model with a blinking component^34^. A summary of these results (see SI5) is presented in Table 2. Again, the average diffusion coefficient recorded for Drp1 in cells (9.14 ± 0.18 μm^2^/s) corresponds to the value predicted using Eq. 1 (9.1 ± 0.5 μm^2^/s, Table 1). Similarly to K668E-Drp1, a relatively high fraction of G363D-Drp1 freely diffuses in cytosol (on average 67% of diffusing objects, Table 2), which is consistent with works indicating limited membrane affinity of G363D-Drp1 mutant^32^.

C505A-Drp1 is a dimer, which is reported in turn to participate in mitochondrial fission^35^. Data obtained for EGFP-C505A-Drp1 were analysed analogously to EGFP-G363D-Drp1 (see SI6), and results are summarized in Table 2. In cytosol, free diffusion of its dimeric form was detected (8.85 ± 0.12 μm^2^/s, corresponding to predicted 9.1 ± 0.5 μm^2^/s). The fraction of freely diffusing protein is much smaller than in the case of G363D-Drp1 mutant (amplitude of 0.44 comparing to 0.67 for G363D-Drp1, see Table 2). This is consistent with work of Macdonald *et al*.^35^: C505A-Drp1, involved in mitochondrial fission, and its exhibited affinity to the mitochondrial membrane. Results obtained for K668E-Drp1, G636D-Drp1 and C505A-Drp1 support the length-scale dependent η_eff_ model applied for the HeLa cell cytosol (Fig. 2). The diffusion coefficients of the monomer and dimer of Drp1 corresponded to the values predicted by Eq. 1. This confirms that η_eff_ of HeLa cytosol is length-scale dependent and our Eq. 1 can be used to describe rheological characteristics of the cellular interior.

### Wild type Drp1 exhibits an equilibrium between dimeric and tetrameric form in cytoplasm

Detailed information on the analysis of FCS data for EGFP-Drp1(wt) is presented in SI7. Briefly, a two component model (as in case of Drp1 mutants) resulted in a poor fit and the value of diffusion time was in-between those expected for the dimer and tetramer. This suggested that there is a mixture of dimer and tetramer in the cytosol (see SI7)^36^. Therefore, fitting was performed using three-component model with diffusion times fixed as values expected for dimer (9.1 ± 0.5 µm^2^/s) and tetramer (5.7 ± 0.4 µm^2^/s), derived from Eq. 1 and calibration coordinates. The amplitude of each component was a free parameter in fitting (see SI7). Table 2 summarizes the results. According to the results, approx. 50% of the signal corresponds to cytosolic fraction of Drp1. Average amplitudes of dimer and tetramer in cytosol were 0.34 and 0.15 respectively. In-depth analysis of the amplitudes of FCS autocorrelation curves led to a conclusion that all oligomers detected by FCS exhibit the same brightness, which is equal to one EGFP molecule (our reasoning is presented in SI8)^36–38^. It follows that FCS amplitudes correspond to actual concentrations and that the average relative quantity of dimers to tetramers in cytosol is 7:3.

### FCS data enables quantification of K_D_ values *in vivo*

**Figure 4:**
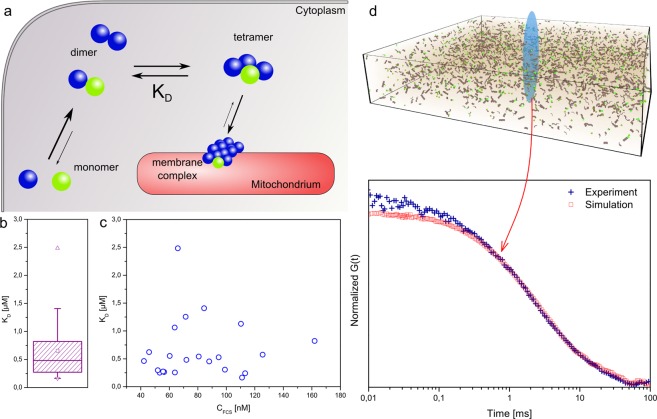
(**a**) Proposed mechanism of Drp1 oligomerization used for K_D_ determination. First step is dimerization - binding of monomers to form dimers. Dimerization is considered to be much faster than a timescale of FCS experiment. Second step is binding of two dimers to form a tetramer. Both dimers and tetramers can be detected in EGFP-Drp1 (wt) expressing cells, so K_D_ of this process can be calculated from FCS data. Next step is binding of tetramers to mitochondrial membrane to form membrane complex. Specific membrane binding cannot be quantified using obtained data, and it cannot be distinguished from unspecific events. Green monomers represent molecules tagged with EGFP (only one subunit is tagged in each oligomer, see SI8). (**b**) A box plot of K_D_ of Drp1 tetramerization obtained in EGFP-Drp1 (wt) expressing cells. Box represents 25, 50 and 75 percentiles, and square (▫) represents an average value. (**c**) The K_D_ plotted against EGFP-Drp1 (wt) expression level. No correlation between these two values can be observed. (**d**) Simulations based on a model concluded from our experiments led to autocorrelation curves perfectly fitting experimental data. Discrepancies for t < 0.1 ms result from EGFP blinking, which was not simulated.

Coexistence of dimers and tetramers in cytosol indicates that the process of Drp1 oligomerization can be divided into three steps: (1) formation of dimers from monomers, (2) formation of tetramers from dimers, and (3) formation of membrane complexes (see Fig. 4a). Quantities of dimers and tetramers in cytosol can be determined with FCS, and the equilibrium constant of tetramer formation can be calculated using these data. The formation of dimers cannot be quantified due to a lack of detectable monomers in samples. Therefore we can assume that dimerization is a fast process, much faster than the timescale of FCS experiments. The third component is a mixture of species forming membrane complexes and species unspecifically bound to random intracellular components. These two populations cannot be distinguished based on diffusion times, so kinetics of membrane complex formation cannot be calculated using this method.

K_D_ is defined as a “dissociation equilibrium constant” of a protein-protein complex^39^. In the case of our experiments, K_D_ is calculated according to Eq. 2:

2 $${K}_{D}=\frac{{C}_{FCS}}{p}\cdot \frac{{{A}_{dimer}}^{2}}{{A}_{tetramer}}$$

where C_FCS_ is concentration of detected molecules (reflecting expression level of tagged Drp1); p is a fraction of tagged Drp1 to overall Drp1 amount; and A_dimer_, A_tetramer_ refer to amplitudes of dimer and tetramer, respectively. The derivation of Eq. 2 is detailed in SI9, made possible by the following assumptions: (1) an equilibrium between dimers and tetramers, (2) an excess of endogenous, untagged Drp1, (3) the existence of a direct proportion between concentrations of molecules and FCS amplitudes^37^, and (4) a lack of large species contribution to the equilibrium between dimers and tetramers. K_D_ was calculated for each cell separately and results ranged between 0.16 and 2.5 μM, with an average of 0.7 ± 0.5 μM (see Fig. 4b). These K_D_ values correspond to moderate-high affinity of dimer-dimer interactions, which could have been expected for this reaction (to our best knowledge, there are no reference experiments providing the strength of this interaction *in vitro*).

The high variety of results was also taken into consideration. We plotted K_D_ values against concentration of fluorescent molecules detected in the cells (Fig. 4c). The plot shows no trend - K_D_ values are distributed randomly, without any link to EGFP-Drp1 expression levels. This lack of trend indicates that the overall concentration of Drp1 molecules does not affect the K_D_. This confirms our assumption that experiments were performed in equilibrium conditions. Moreover, despite a relatively large population heterogeneity, dimer-dimer affinity is high (K_D_ < 1 μM) in a majority of cells (>75%). We used Monte Carlo simulations as an alternative method to confirm our approach (Fig. 4d). Using physical results from our interpretation of FCS data as an input, the simulator (see SI10) returned a set of autocorrelation curves which matched experimental curves (Fig. 4d). Additionally, a fit of the simulated curves resulted in average amplitudes of 0.36 ± 0.06, 0.16 ± 0.11 and 0.48 ± 0.06 for dimers, tetramers and large fraction respectively (comparing to 0.34 ± 0.09, 0.15 ± 0.05 and 0.51 ± 0.1 from cell experiments). This agreement validates the proposed strategy of data interpretation.

## Discussion

In this work, we discussed quantitative interpretation of diffusion coefficients recorded by means of FCS in cytosol of living cells. Our idea aiming to determine the size of protein complexes in cytosol first required the quantification of cytosolic hydrodynamic drag. We concluded that apart from spatial heterogeneity, length-scale dependent heterogeneity is also pronounced in cytosol (Fig. 2). Thorough determination of *in vivo* diffusion coefficients of probes with known hydrodynamic radii revealed that cytosolic hydrodynamic drag increases with probe size. This dependency was described in Eq. 1. With this conclusion, FCS allows us to determine the size of the probe freely diffusing in the cytosol. Our findings were applied to the problem of determining the state of protein oligomerization *in vivo*. We chose Drp1 as a model oligomerizing protein, which is known to form dimers, tetramers and bigger complexes, but the exact location of each of those forms (membrane-bound or cytosolic) remains unclear. We addressed this issue with our FCS-based method of *in vivo* molecule size determination. First, we investigated Drp1 mutants unable to form bigger oligomers (mutations: K668E, G363D and C505A). FCS measurements of these forms agreed with our model of cytosolic η_eff_; obtained diffusion coefficients corresponded to the predicted values. Wild type Drp1 was examined next. In cytosol of cells with fully functional Drp1 two types of oligomers were detected: dimers and tetramers. Based on all obtained data, we proposed a scenario of Drp1 oligomerization (Fig. 4a). Independent computer simulations were performed base on the proposed mechanism which gave results matching experimental data. As explained earlier, experimental data provided quantitative information on the number of molecules of each oligomer in the sampled cytosol. This enabled the calculation of tetramers’ K_D_, which gave an average of 0.7 μM.

To conclude, we present an FCS-based method to quantify protein interactions in living cells. A major advantage of our approach (compared to analogous FRET experiments) is the requirement for only one fluorescently labelled probe, reducing the number of steps needed to prepare an experiment. Moreover, invasive microinjection can be avoided, allowing more mild experimental conditions and more reliable results. Another important issue is probing the equilibria of native, endogenous proteins. We used protein oligomerization as an example to validate our approach, but the range of possible applications is extensive. Precise interpolation of cytosolic hydrodynamic drag enables detection of the hindered motion of biomolecules. Therefore, FCS-based quantification of molecular interactions in living cells can be used for K_D_ determination not only of protein oligomerization, but also protein-ligand binding, or drug-target interactions. We predict that studies of biochemical reaction kinetics *in vivo* will be crucial for understanding and tackling pathological changes in cellular metabolism.

## Methods

### Plasmid preparation

The cDNA coding for isoform 1 (transcript variant 1) of Drp1 was obtained from Origene (RG221708). The cDNA was amplified by PCR using primers (for: AGCTTCGAATTCTATGGAGGCGCTAATTCCTGTCATAAACAAGCTC, rev: CTGAAATCCGGGAGACTCATCTTTGGTGAGGATCCACCGGA) and subcloned to the pmEGFP-C1 vector using restriction enzymes EcoRI and BamHI (pmEGFP-C1 was a gift from Benjamin Glick (Addgene plasmid #36412)). The mutants of K668E, G363D and C505A of Drp1 were obtained by QuikChange site-directed mutagenesis^40^ and confirmed by DNA sequencing. Primers used for obtaining Drp1 mutants were following: K668, for: GTGCCAGAGGCAGTAATGCATTTTTTGGTT, rev: TACTGCCTCTGGCACACTGTCTTGAATATT; G363D, for: TGCGGTGATGCTAGAATTTGTTATATTTTCCATGAG, rev: TCTAGCATCACCGCATAGCTCCGAA; C505A, for: GCTGATGCTGCTGGGCTAATGAACAATAATAT, rev: TAGCCCAGCAGCATCAGCAAAGTCTG. Plasmids were amplified using endotoxin free maxi-prep kits (Sigma-Aldrich). GFP-Apoferritin plasmid was obtained using plasmid encoding ferritin heavy chain 1 (FTH1, Origene) and pmEGFP-C1. Sequence encoding FTH1 was subcloned into pmEGFP-C1 vector using restriction enzymes BglII and EcoRI and primers for FTH1 amplification: for: GACTCAGATCTTCCGGCGCAGCAGCAGGTGGAGGTTCGGGTGGAGGTAGCGGTGGAGGTATGACGACCGCGTCCACC, rev: ACTGCAGAATTCTTAGCTTTCATTATCACTGTCTCCCAGGGTG.

### Cell culture, transfection and microinjection

HeLa cells, Kyoto strain, were obtained from Jan Ellenberg (EMBL Heidelberg) with the permission from Shuh Narumiya (Department of Pharmacology, Kyoto University Graduate School of Medicine). Cells were cultured as monolayers using Dulbecco’s modified Eagle’s medium (DMEM, Institute of Immunology and Experimental Technology, Wrocław, Poland) supplemented with 10%_vol_ fetal bovine serum, penicillin (100 mg/ml) and streptomycin (100 mg/ml) (Sigma-Aldrich). Cells were maintained at 37 °C in a 5% CO_2_ humidified atmosphere. Passage was performed using 0.25% Trypsin-EDTA solution (Sigma-Aldrich) and phosphate-buffered saline (PBS, Sigma-Aldrich). For transfection cells were grown on 8-chamber cover glass Lab-Tek® slide to approx. 30% of confluence. Transfection was performed using JetPRIME® reagent (Polyplus Transfection) according to manufacturer’s protocol. FCS experiments were performed 24 hours after transfection. For microinjection cells were grown on 35 mm glass bottom dishes to approx. 30% of confluence. Microinjection was performed by Femtojet® system (Eppendorf), with glass capillaries of diameters <1 μm, prepared using micropipette puller (P-1000, Sutter Instrument). The concentrations of injected solutions were in the range of 5–40 μM (in PBS). Approx. 1000 cells were injected per experiment with settings: injection pressure 160 hPa, injection time 0.2 s and compensation pressure 30 hPa. FCS measurements were performed 1 h after microinjection.

For experiments in cell lysates, transfected cells were prepared according to standard passage. After the tripsinization cells were suspended in 1 ml of fresh media. Cell suspension was centrifuged (3500 rpm; 5 min), washed with 1 ml of PBS and finally suspended in 0.5 ml of PBS. Then 20 µl of non-denaturing cell lysis buffer was added^41^. The buffer contained 10 mM imidazole (Fluka, USA), 0.5 M sodium chloride (Fluka, USA), 1% Triton X 100 (Sigma-Aldrich, USA), 0.2 mM sodium ortho-vanadate (Sigma-Aldrich, USA) and 0.2 mM phenylmethylsulfonyl fluoride (PMSF; Fluka, USA) dissolved in 100 ml of deionized water. After 2 min of incubation the samples were ready for the measurements. For each cell lysate (containing different EGFP-Drp1 variant) a series of 10 measurements each lasting 30 s was performed.

### Fluorescent tracers

PEG-coated fluorescent silica nanoparticles filled with Rhodamine B were custom-synthetized for the purpose of this research by Siliquan (https://siliquan.com/). TRITC-labelled dextrans (Sigma-Aldrich) and fluorescent nanoparticles where introduced into cytosol via microinjection. Calcein-AM (Sigma-Aldrich) was spontaneously uptaken by cells. EGFP and EGFP-tagged apoferritin were expressed after transfection with appropriate plasmids. Hydrodynamic radii of proteins were calculated using HydroPro software.

### Fluorescence correlation spectroscopy

FCS was performed using setup based on confocal microscope (Nikon Eclipse TE2000U, Nikon, Japan) coupled with Pico Harp 300 FCS equipment (PicoQuant, Germany). Measurements were performed in a climate chamber (Okolab, Italy) providing temperature control (36 ± 0.5 °C) and atmosphere of required composition and humidity. He-Ne laser (543 nm) and pulsed diode laser (481 nm) were used as light sources. Power were kept in the ranges of 5–20 μW for 543 nm laser and 1–5 μW for 481 nm laser. Probes were observed and measured through a 60 × (N.A. 1.2) objective with water immersion. Fluorescent signal for FCS was collected by Single Photon Avalanche Diodes (MPD and PerkinElmer). Each experiment was preceded by a calibration using Rhodamine B (λ_ex_ = 543 nm, Sigma-Aldrich) dissolved in a solution of 2.5%_wt_ glucose in PBS^36^. The objective correction collar was adjusted to provide optimal FCS read-out, and RhoB diffusion was used for precise characterization of focal volume size and shape. These values were further used for calculations of concentrations of measured molecules. For FCS experiments on cells, cell culture medium was replaced with DMEM without phenol red (Sigma-Aldrich) to avoid background signal. The detection volume was positioned in a cytoplasmic area of a cell using imaging mode of the microscope.

### Data processing, fitting and modelling

SympPhoTime software (PicoQuant, Germany) was used for time traces acquisition and autocorrelation curves calculation. Fitting was performed according to appropriate diffusion model using Gnuplot 5.0 software. FCS data were fitted using Eq. 3,

3 $$G(\tau )=\frac{1}{N}{\sum }_{i=1}^{n}\,{A}_{i}\frac{1}{1+{(\frac{\tau }{{\tau }_{Di}})}^{\alpha }}\frac{1}{\sqrt{1+\frac{1}{{\kappa }^{2}}{(\frac{\tau }{{\tau }_{Di}})}^{\alpha }}},$$

where N stands for the average number of fluorescent probes inside the focal volume, τ_Di_ is the average time of diffusion of an *i* probe across the focal volume, A_*i*_ corresponds to the amplitude of the i^th^ component, κ is the aspect ratio of the focal volume (measured during calibration), and α is an anomalous exponent (α = 1 for normal 3D diffusion, and α < 1 for anomalous subdiffusion). During fitting the value of the amplitude of the fast component was constrained between 0–1. To fulfil the assumption about blinking of the GFP proteins we used the model including triplet state kinetics with the characteristic time constrained in the range from 0.1 to 0.3 ms^34^. Monte Carlo simulations were performed using Mcell simulator^42–44^ supported by CellBlender 1.1 and FERNET Toolkit^45^.

## Supplementary information

Supplementary Information (SI)
