# Supplementary material for: Determination of oligomerization state of Drp1 protein in living cells at nanomolar concentrations
Source: Sci Rep. 2019 Apr 11;9:5906. doi: 10.1038/s41598-019-42418-0 (PMC6459820; doi:10.1038/s41598-019-42418-0)
Supplement: Supplementary file 1 — Supplementary Information (SI) [file 41598_2019_42418_MOESM1_ESM.pdf]

Supplementary Information

for

Determination of oligomerization state of Drp1 protein in living cells at nanomolar concentrations

Karina Kwapiszewska[1,*], Tomasz Kalwarczyk[1], Bernadeta Michalska[2], Krzysztof Szczepański[1], Jędrzej Szymański[2], Paulina Patalas-Krawczyk[2], Tomasz Andryszewski[1], Michalina Iwan[1], Jerzy Duszyński[2], & Robert Hołyst[1,*]

[1]*Institute of Physical Chemistry, Polish Academy of Sciences, Kasprzaka 44/52, 01-224 Warsaw, Poland;*

[2]*Nencki Institute of Experimental Biology, 3 Pasteur St, 02-093 Warsaw, Poland*

**corresponding author: karina.kwapiszewska@gmail.com, robert.holyst@gmail.com*

SI1. Determination of hydrodynamic radii of tracers

TRITC labelled dextrans and custom-synthetized, PEG-coated **silica nanoparticles** were dissolved in DI water to final concentrations of approx. 10nM and were further used for FCS measurement. The autocorrelation curves were fitted using anomalous diffusion model*, without triplets. Diffusion times were calculated according to equation SI1.1.

$$D = \frac{{\omega_{xy}}^2}{4\,\tau_D}, \qquad \text{(SI1.1)}$$

Where D is diffusion coefficient [m^2/s], τ_D is diffusion time [s] resulted from FCS autocorrelation curve fitting, and ω_{xy} is a radius of xy plane of a focal volume [m]. Parameter ω_{xy} was determined for each experiment separately during calibration step. Calibration was performed using 10nM Rhodamine B in water.

The diffusion coefficients were used for hydrodynamic radii (r_p) calculation, according to Stokes-Sutherland-Einstein relation (equation SI1.2).

$$r_p = \frac{k\,T}{6\pi\,\eta\,D}, \qquad \text{(SI1.2)}$$

Where k is the Boltzman constant, T refers to temperature at which measurement was conducted [K], and η is the viscosity of water [Pa·s] in given temperature T.

Hydrodynamic radius of **Calcein** was obtained from literature (ref. 27).

EGFP and EGFP tagged **apoferritin** were modelled using HydroPro software.

All hydrodynamic radii for tracers were set together in Table SI1.

Table SI1.1. Summary of hydrodynamic radii of tracers used for determination of cytoplasmic hydrodynamic drag.

Tracer	r_p [nm]	method
Calcein	0.65	Literature (ref. 28)
Dextran 4.4 kDa	1.3	FCS measurement
Dextran 20 kDa	3.8	FCS measurement
Dextran 40 kDa	4.9	FCS measurement
Dextran 65-85 kDa	5.6	FCS measurement
Dextran 155 kDa	8.6	FCS measurement
EGFP	2.3	HydroPro modelling
EGFP-apoferritin	9.4	HydroPro modelling
Nanoparticles 1	21	FCS measurement
Nanoparticles 2	34	FCS measurement

* According to dextrans' and nanoparticles' polydispersity, see ref. 36.

SI2. Determination of diffusion coefficients of tracers in the cytoplasm of HeLa cells

Dextrans and nanoparticles were introduced into cells by microinjection. Proteins (EGFP and EGFP-apoferritin) were expressed after transfection with proper plasmids. Calcein was introduced to cell culture medium as calcein-AM and spontaneous cellular uptake occurred within minutes.

For each experiment, size of a focal volume (ω_{xy}) was determined during calibration.† Cells filled with tracers were placed in experimental setup and focal volume was positioned in cytoplasmic area. FCS measurements were performed for 0.25-10 minutes, depending on a tracer‡. Each tracer was measured in at least 15 cells from at least two different inocula. Diffusion coefficients were calculated according to equation SI1.1 and displayed in table SI2.2. According to the Stokes-Sutherland-Einstein relation, equation SI2.1 can be derived:

$$\frac{\eta_{eff}}{\eta_0} = \frac{D_{water}}{D_{cytosol}}, \qquad \text{(SI2.1)}$$

Using equation SI2.1, relative hydrodynamic drag (η_{eff}/η_0) was calculated for each probe.

Table SI2.1. Determination of relative hydrodynamic drag of HeLa cells' cytosol at 36°C.

Tracer	r_p [nm]	D [µm2/s]*	η_{eff}/η_0 **
Calcein	0.65	281 ± 99	1.99 ± 0.72
Dextran 4.4 kDa	1.3	79 ± 21	3.28 ± 0.96
Dextran 20 kDa	3.8	28.0 ± 4.5	3.12 ± 0.48
Dextran 40 kDa	4.9	13.8 ± 3.0	4.89 ± 0.94
Dextran 65-85 kDa	5.6	11.0 ± 4.0	5.9 ± 2.0
Dextran 155 kDa	8.6	4.9 ± 2.0	8.2 ± 1.9
EGFP	2.3	56 ± 11	2.65 ± 0.60
Apoferritin	9.4	5.5	6.2
Nanoparticles 1	21	1.38 ± 0.28	11.7 ± 2.3
Nanoparticles 2	34	0.75 ± 0.21	13.4 ± 2.6

* averaged diffusion coefficient from N≥15 cells, measured in cytosol by FCS
** relative hydrodynamic drag of cytosol for a given probe, related to pure water.
Errors refer to standard deviations

SI3. HydroPro calculations

Diffusion coefficients in water D_0 at temperature of 37° C and viscosity of 0.69 mPas were calculated using HydroPro software (Ortega2011) based on the available structural data (RCSB Protein Data Bank and EMDataBank). The size of a given oligomer was estimated as hydrodynamic radius, r_p, calculated from respective D_0 using Stokes-Sutherland-Einstein relation, (equation SI1.2). The protein data bank (PDB) structures of Drp1 (4BEJ), GFP (1EMA) and ferritin (4YKH) were used to build models of Drp1 and GFP-Drp1 oligomers as well as model molecules

† Calibration was performed using 10 nM Rhodamine B in 2.5%$_{w/w}$ glucose in PBS (see reference 36).
‡ Acquisition time was dependent on a fluorophore photostability and expected diffusion time of a tracer.

(GFP, ferritin). The structure of GFP-Drp1 was created in Chimera software and saved in pdb format for calculations in HydroPro software. The four Drp1 molecules (monomers) present in the 4BEJ asymmetric unit of 4BEJ structure were used as one of the models of the tetramer. Biological assembly was used as a model of dimer and one selected Drp1 molecule from a dimer was a model of the monomer.

SI4. Drp1 analysis in cell – K668E mutant (monomer)

FCS data was acquired in a cytosol of cells transfected with EGFP-K668E-Drp1 protein. 10 measurements were performed per cell and acquisition time of each curve was 30 s. The FCS autocorrelation curves were normalized and summarized for each cell, and then fitted using two component model. Diffusion coefficients for each component were calculated using equation SI1.1, where τ_D was a value obtained from fitting and ω_{xy} was determined during calibration (separately for each experiment). Results were summarized in table SI4.1.

Table SI4.1. Results of fitting of EGFP-K668E-Drp1 data obtained in cytosol in 36°C.

**Cell**	**$D_{monomer}$ [µm^2/s]**	**$A_{monomer}$**	**D_{large} [µm^2/s]**	**A_{large}**
I	15.61	0.93	0.80	0.07
II	15.70	0.84	1.36	0.16
III	15.64	0.90	0.42	0.10
IV	15.70	0.85	1.15	0.15
V	15.59	0.88	0.28	0.12
VI	15.53	0.78	1.45	0.22
VII	15.36	0.63	1.41	0.37
VIII	15.70	0.70	3.48	0.30
IX	15.70	0.76	1.41	0.24
X	15.42	0.87	0.99	0.13
XI	15.42	0.79	1.18	0.21
XII	15.47	0.67	1.84	0.33
XIII	15.70	0.93	0.34	0.07
XIV	15.47	0.72	1.57	0.28
XV	15.52	0.81	1.37	0.19
XVI	15.65	0.87	0.78	0.13
XVII	15.47	0.92	0.45	0.08
XVIII	15.52	0.76	1.22	0.24
XIX	15.37	0.68	1.46	0.32
XX	15.32	0.73	1.39	0.27
**Average**	**15.54**	**0.80**	**1.22**	**0.20**
Error	0.13	0.09	0.68	0.09

A_{dimer} and A_{large} are FCS amplitudes obtained for each component.

Results for bigger oligomers indicate existence of EGFP blinking phenomenon (see SI5, SI6 and SI7 sections). It was expected, that blinking should also occur in EGFP-K668E-Drp1 samples. However, blinking time (approximately 150 μs) was close to monomer diffusion time (approx.. 550 μs, varying according to calibration settings). These two times could not be distinguished by fitting and thus simple two component model was used.

SI5. Drp1 analysis in cell – G363D mutant (dimer)

FCS data was acquired in a cytosol of cells transfected with EGFP-G363D-Drp1 protein. 10 measurements were performed per cell and acquisition time of each curve was 30 s. The FCS autocorrelation curves were normalized and summarized for each cell, and then fitted using two component model with additional component for EGFP blinking (see reference 34). Diffusion coefficients for each component were calculated using equation SI1.1, where τ_D was a value obtained from fitting and ω_{xy} was determined during calibration (separately for each experiment). Results were summarized in table SI5.1.

Table SI5.1. Results of fitting of EGFP-G363D-Drp1 data obtained in cytosol in 36°C.

Cell	D_{dimer} [μm²/s]	A_{dimer}	D_{large} [μm²/s]	A_{large}	$\tau_{blinking}$ [ms]	$A_{blinking}$
I	8.99	0.47	1.73	0.53	0.11	0.16
II	9.05	0.54	1.25	0.46	0.11	0.14
III	8.99	0.56	0.46	0.44	0.13	0.14
IV	9.20	0.60	0.83	0.40	0.16	0.12
V	8.98	0.84	0.07	0.16	0.10	0.14
VI	8.88	0.75	1.05	0.25	0.13	0.16
VII	9.25	0.78	0.78	0.22	0.18	0.17
VIII	9.04	0.66	0.62	0.34	0.19	0.17
IX	9.09	0.69	0.98	0.31	0.12	0.15
X	9.20	0.68	0.31	0.32	0.15	0.14
XI	9.12	0.68	1.12	0.32	0.14	0.07
XII	9.38	0.64	1.01	0.36	0.15	0.12
XIII	9.10	0.74	0.93	0.26	0.12	0.11
XIV	8.84	0.52	0.85	0.48	0.11	0.13
XV	9.45	0.72	0.33	0.28	0.14	0.14
XVI	9.50	0.72	1.10	0.28	0.19	0.16
XVII	9.27	0.86	0.05	0.14	0.14	0.09
XVIII	9.17	0.64	0.24	0.36	0.17	0.17
**Average**	**9.14**	**0.67**	**0.76**	**0.33**	**0.14**	**0.14**
Error	0.18	0.10	0.44	0.10	0.03	0.03

A_{dimer}, $A_{bounded}$ and A_{large} are FCS amplitudes obtained for each component.

SI6. Drp1 analysis in cell – C505A mutant (dimer with fission functionality)

FCS data was acquired in a cytosol of cells transfected with EGFP-C505A-Drp1 protein. 10 measurements were performed per cell and acquisition time of each curve was 30 s. The FCS autocorrelation curves were normalized and summarized for each cell, and then fitted using two component model with additional component for EGFP blinking (see reference 34). Diffusion coefficients for each component were calculated using equation SI1.1, where τ_D was a value obtained from fitting and ω_{xy} was determined during calibration (separately for each experiment). Results were summarized in table SI6.1.

Table SI6.1. Results of fitting of EGFP-C505A-Drp1 data obtained in cytosol in 36°C.

Cell	D_{dimer} [μm²/s]	A_{dimer}	D_{large} [μm²/s]	A_{large}	$\tau_{blinking}$ [ms]	$A_{blinking}$
I	8.85	0.34	0.96	0.66	0.18	0.05
II	9.14	0.23	0.61	0.77	0.15	0.05
III	8.78	0.53	0.83	0.47	0.10	0.09
IV	8.88	0.37	1.32	0.63	0.10	0.06
V	8.80	0.52	1.12	0.48	0.10	0.10
VI	8.78	0.40	1.37	0.60	0.15	0.05
VII	8.70	0.65	1.01	0.35	0.10	0.11
VIII	8.85	0.51	1.16	0.49	0.10	0.11
**Average**	**8.85**	**0.44**	**1.05**	**0.56**	**0.12**	**0.08**
Error	0.12	0.12	0.23	0.12	0.03	0.03

A_{dimer}, A_{large} and $A_{blinking}$ are FCS amplitudes obtained for each component.

Content of freely diffusing dimer is 44% (on average). This is lower than in G363D mutant (67%), which indicates higher membrane affinity of C505A-Drp1.

SI7. Drp1 analysis in cell – wild type

FCS data was acquired in a cytosol of cells transfected with EGFP-Drp1 (wild type) protein. 10 measurements were performed per cell and acquisition time of each curve was 30s. The FCS autocorrelation curves were normalized and summarized for each cell, and then fitted. First model of choice was analogous for previous experiments - two component model with additional component for EGFP blinking. Diffusion times obtained from this fitting were longer than expected for dimer and shorter than expected for tetramer (see Fig. SI7.1). Our previous work indicate, that one diffusion time obtained from FCS can be resultant from two or more diffusing species of comparable sizes (see reference 36). The concept for Drp1 was illustrated in Fig. SI7.2. Experimental data was fitted with three-component model with amplitudes and diffusion time of bound component as parameters. According to limitations of fitting algorithm, diffusion times for dimer and tetramer should have been fixed. We justify this approach by the results achieved for EGFP-K668E-Drp1, EGFP-G363D-Drp1, EGFP-C505A-Drp1, which confirmed our predictions. Diffusion times were calculated (Eqn. SI1.1) using expected diffusion coefficients of oligomers

(see Table 1, main text) and ω_{xy} calibrated for each data set. Results of the fitting were summarized in Table SI7.1.

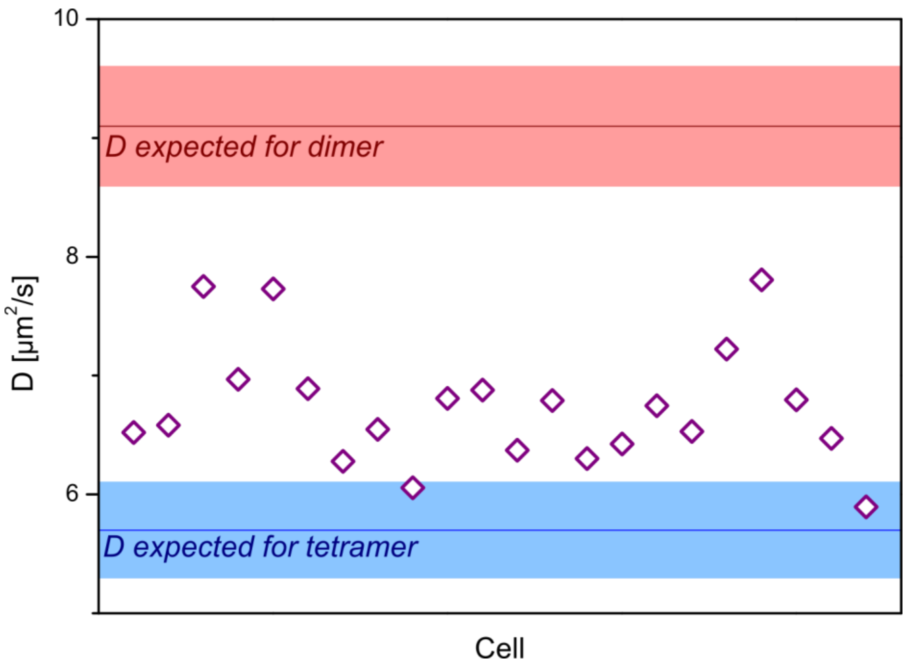

Figure SI7.1. Results of fitting of autocorrelation curves of EGFP-Drp1 (wt) in HeLa cells, using 2-component free diffusion model. Diamonds represent diffusion coefficients calculated form fitting. Majority of the results cannot be assigned to dimer nor to tetramer. Thus, mixture of dimer and tetramer was suggested.

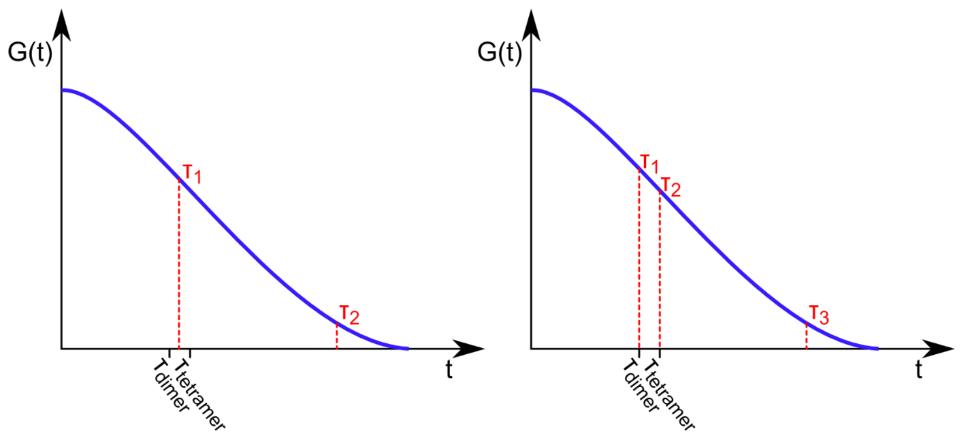

Figure SI7.2. Principle of detection of oligomers mixture in a cytosol. Two-component fit (left panel) results in τ_1 (corresponding to cytosolic fraction of a protein) laying between values expected for dimer and tetramer. Thus, in the next step, three-component model was applied to split diffusion times for dimer and tetramer (right panel). In case of Drp1 there is always one very long component in cells, thus at least 2-component fit is necessary.

Table SI7.1. Results of fitting of EGFP-Drp1(wt) data obtained in cytosol in 36°C.

Cell	A_{dimer}	$A_{tetramer}$	D_{large} [μm²/s]	A_{large}	$\tau_{blinking}$ [ms]	$A_{blinking}$
I	0.33	0.15	1.01	0.52	0.12	0.08
II	0.25	0.14	0.81	0.61	0.16	0.04
III	0.33	0.07	1.77	0.60	0.12	0.10
IV	0.56	0.19	0.62	0.25	0.11	0.14
V	0.50	0.12	0.21	0.39	0.11	0.12
VI	0.31	0.14	1.01	0.55	0.13	0.10
VII	0.30	0.08	0.71	0.62	0.14	0.07
VIII	0.21	0.12	0.91	0.67	0.18	0.06
IX	0.35	0.29	0.65	0.37	0.15	0.05
X	0.33	0.12	1.43	0.55	0.14	0.06
XI	0.31	0.22	0.39	0.47	0.15	0.08
XII	0.21	0.19	0.78	0.59	0.14	0.05
XIII	0.37	0.17	1.36	0.46	0.13	0.04
XIV	0.27	0.18	0.80	0.56	0.11	0.09
XV	0.23	0.16	0.60	0.61	0.14	0.03
XVI	0.22	0.11	0.90	0.67	0.19	0.09
XVII	0.32	0.13	0.66	0.54	0.17	0.06
XVIII	0.46	0.06	0.57	0.48	0.11	0.13
XIX	0.45	0.11	1.22	0.44	0.13	0.17
XX	0.40	0.16	0.70	0.44	0.10	0.14
XXI	0.39	0.20	0.70	0.41	0.13	0.15
XXII	0.37	0.17	0.52	0.46	0.12	0.14
**Average**	**0.34**	**0.15**	**0.83**	**0.51**	**0.13**	**0.09**
Error	0.09	0.05	0.35	0.10	0.02	0.04

SI8. Brightness of oligomers

FCS autocorrelation curve *G(τ)* depends *inter alia* on a square of molecular brightness of a probe (see reference 36). Therefore, if a mixture of a probes is considered, brightness should be taken into account in analysis of the probes' relative quantities. In case of Drp1 dimer and tetramer, if each of monomers consist of an EGFP tag, then tetramer should be twice as bright as dimer. Thus, tetramer contribution in FCS signal would be 4-times bigger than it would result from its concentration.

Plasmid coding EGFP-Drp1 was introduced into native cells, expressing their endogenous Drp1 levels. According to Western blot analysis (see reference 27), total concentration of endogenous Drp1 in HeLa Kyoto cells is 0.6 mM (counted per monomer). Concentration of EGFP-tagged protein can be calculated basing on FCS autocorrelation curve amplitude, which is equal to N^{-1}, where N is a number of fluorescent objects in a focal volume. FCS autocorrelation curves of EGFP-Drp1(wt) before normalization were fitted with a three component diffusion model. Obtained N

values were used for calculation of fluorescent objects concentrations (C), according to equation SI8.1.

$$C = \frac{N}{N_A \, \pi \, h \, \omega_{xy}^2} \quad \text{SI8.1}$$

where N_A is Avogadro constant, h is height of detection volume (assumed to be equal to $1 \cdot 10^{-6}$m, constant for all measurements) and ω_{xy} is a radius of a focal volume considered for each experiment separately. Geometry of focal volume in a cell is presented in Fig. SI8.1. Obtained C values ranged between 42 to 125 nM. One should be aware, that these values could be underestimated, if a third, large component would come from bigger and much brighter oligomers (see reference 37). Data obtained for EGFP-K668E-Drp1 and EGFP-G363D-Drp1 (mutants unable to form bigger oligomers) indicate, that amplitudes resulting from unspecific binding of Drp1 to intracellular components can reach 0.33 (on average, see tables SI4.1 and SI5.1). Amplitude for large components for EGFP-Drp1 (wt) was, on average, 0.51 (see table SI7.1), so only approximately 1/3 of the third amplitude would come from bigger oligomers. This would lead to no more than 5% error in determining of N[§]. To support our considerations, we analyzed average diffusion coefficients of large components in each cells and tried to correlate them with apparent concentrations or amplitudes (figure SI8.1). If the large component resulted from bigger and brighter oligomers, increasing size (and brightness) would result in (1) decreasing diffusion coefficient, (2) increasing amplitude, and (3) decreasing apparent concentration. As was shown in figure SI8.2, none of these dependences could have been noticed. Thus, we can assume that large oligomers of bigger brightness were immobile in a timescale of FCS measurements and do not contribute in FCS signal. This assumption is in accordance with the fact, that large Drp1 oligomers are formed on mitochondrial membranes as fission complexes and can be detected as immobile bright spots (see reference 23).

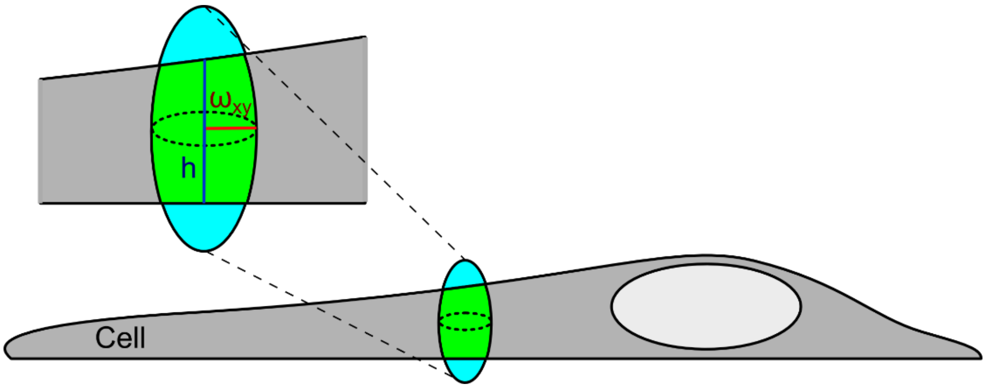

Figure SI8.1. Geometry of a focal volume positioned in a cytoplasm of an adherent cell. Only volume marked as green gives fluorescence signal and its volume can be estimated to the cylinder of radius of ω_{xy} and height of *h*.

§ Calculated following section "FCS for Multiple Diffusing Species" of reference 33.

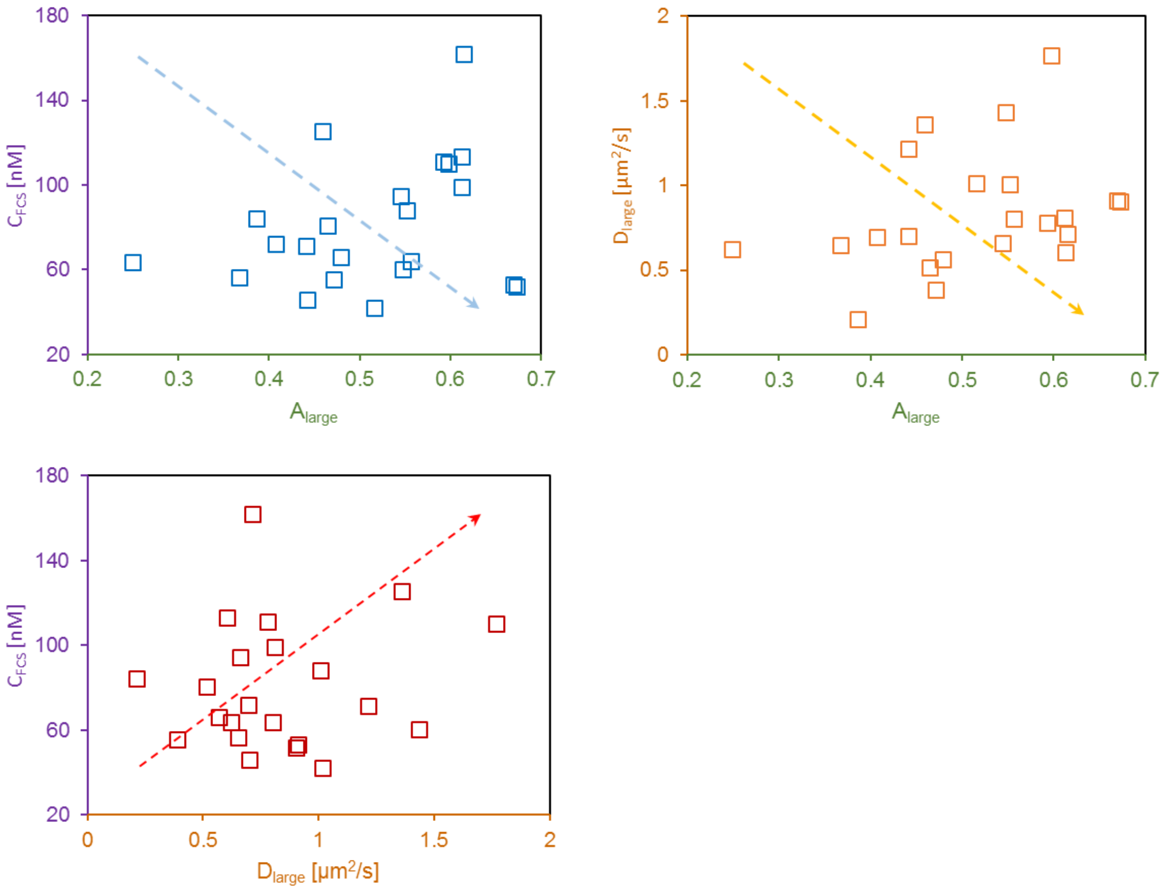

Figure SI8.2. Graphs representing lack of correlations between amplitudes of large objects (A_{large}, green axes), concentrations measured by FCS (C_{FCS}, purple axes) and diffusion coefficients determined for large objects (D_{large}, orange axes). Arrows indicate trends to be expected if brightness of large probes would affect the results. Data points obtained for EGFP-Drp1 (wt).

Amount of Drp1 in transfected cells is a sum of endogenous Drp1 (580 nM, Western blot) and tagged, EGFP-Drp1 expressed from the introduced plasmid. For FCS experiments only cells with low EGFP-Drp1 expression were picked to obtain N optimal for measurements (see figure 3). Concentration of objects measured by FCS (tagged) was in the range of 42 to 125 nM, which gives fraction of EGFP-Drp1 of 7-22% (average 12 ± 4%). Thus, there is an excess of endogenous Drp1 in examined cells. Moreover, steric limitations account for higher probability of EGFP-Drp1 to $Drp1_{(endo)}$ binding, than of two EGFP-Drp1 molecules (analogous for EGFP-dimers and $dimers_{(endo)}$). EGFP dimerization has also low probability as it occurs at higher concentrations (approx. 0.1 mM, see reference 38). Following, on average we can assume, that each dimer or tetramer detected by FCS contains only one EGFP-Drp1 molecule. Going further, considerations concerning large component resulted in conclusion, that molecules of a brightness equal to dimers and tetramers have major contribution to the signal. Concluding, we assume equal brightness of all diffusing objects.** Types of objects present in cytosol of EGFP-Drp1 (wt) expressing cells are presented schematically in Fig. SI8.3.

** Equal to brightness of one EGFP molecule.

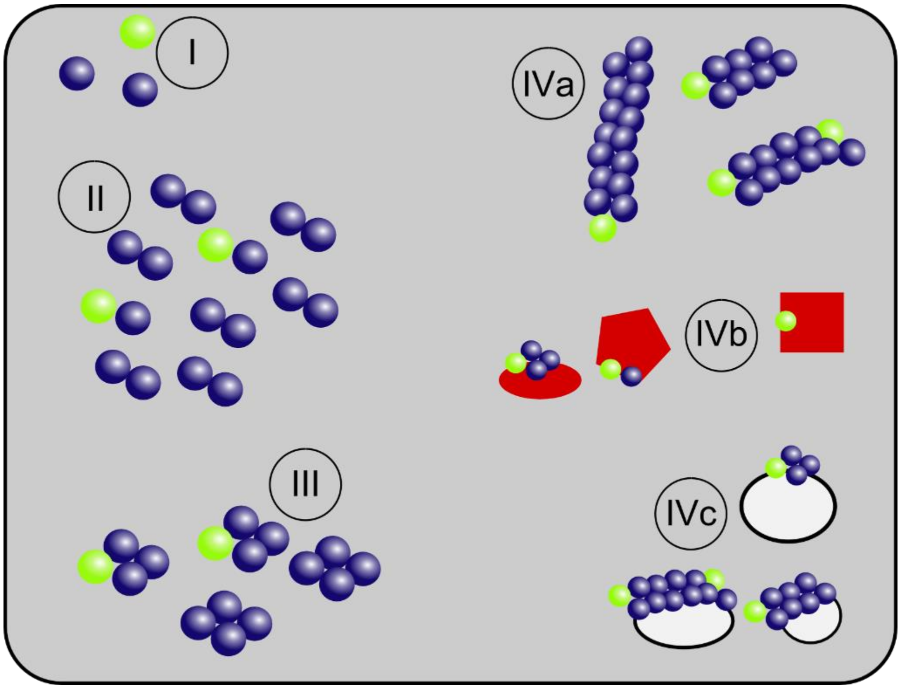

Figure SI8.3. Populations of oligomers present in cytoplasm of EGFP-Drp1 (wt) expressing cells. (I) Freshly synthetized monomers, the amount too small to be detectable by FCS. (II) Dimers, part of which can be detected due to presence of EGFP-tagged monomers (displayed as green spheres). Dimers give contribution to FCS signal with an amplitude A_{dimer}. (III) Tetramers, which contribute to FCS signal with an amplitude $A_{tetramer}$. (IV) Large objects of different types contributing to FCS signal with an amplitude A_{large}. Among large objects, there are at least three possible subpopulations: (IVa) larger oligomers, self-assembled or detached from mitochondria after fission; (IVb) unspecific complexes with other macromolecules, and (IVc) complexes with intracellular membranes.

SI9. K_D determination

K_D was determined for reaction of forming tetramer from dimers (equation SI9.1).

$$D + D \leftrightarrow T \qquad \text{SI9.1}$$

Following the definition (see reference 38), K_D can be calculated according to equation SI9.2.

$$K_D = \frac{[D]^2}{[T]} \qquad \text{SI9.2}$$

Where [D] and [T] are concentrations of dimers and tetramers, respectively. [D] and [T] can be calculated according to equations SI9.3.

$$[D] = \frac{[D^*]}{2\,p}$$
$$[T] = \frac{[T^*]}{4\,p} \qquad \text{SI9.3}$$

Where [D*] and [T*] are concentrations of tagged dimers and tetramers, respectively, and p is a fraction of tagged Drp1 monomers in the cell (calculated as C_{FCS} to C_{total} ratio). [D*] and [T*] can be derived directly from FCS autocorrelation function fitting, according to equation SI9.4.

$$[D^*] = C_{FCS} \cdot A_{dimer}$$
$$[T^*] = C_{FCS} \cdot A_{tetramer} \qquad \text{SI9.4}$$

Combining equations SI9.2, SI9.3 and SI9.4, equation SI9.5 can be derived.

$$K_D = \frac{C_{FCS}}{p} \cdot \frac{A_{dimer}^2}{A_{tetramer}} \qquad \text{SI9.5}$$

K_D was calculated according to equation SI9.5 for each cell separately. Results are presented in table SI9.1.

Table SI9.1. Results of determination of K_D of Drp1 tetramers in cytosol of HeLa cells (36°C)

**Cell**	**C_{FCS} [nM]**	**A_{dimer}**	**$A_{tetramer}$**	**C_{total} [nM]**	**p**	**K_D [μM]**
I	42.2	0.33	0.15	622	0.068	**0.46**
II	99.0	0.25	0.14	679	0.146	**0.31**
III	110.2	0.33	0.07	690	0.160	**1.13**
IV	63.8	0.56	0.19	644	0.099	**1.06**
V	84.4	0.50	0.12	664	0.127	**1.41**
VI	88.0	0.31	0.14	668	0.132	**0.45**
VII	53.2	0.21	0.12	633	0.084	**0.25**
VIII	56.4	0.35	0.29	636	0.089	**0.26**
IX	60.3	0.33	0.12	640	0.094	**0.55**
X	55.6	0.31	0.22	636	0.088	**0.27**
XI	111.0	0.21	0.19	691	0.161	**0.16**
XII	125.5	0.37	0.17	705	0.178	**0.58**
XIII	63.9	0.27	0.18	644	0.099	**0.26**
XIV	113.2	0.23	0.16	693	0.163	**0.24**
XV	51.9	0.22	0.11	632	0.082	**0.29**
XVI	161.9	0.30	0.08	742	0.218	**0.82**
XVII	94.6	0.32	0.13	675	0.140	**0.53**
XVIII	66.0	0.46	0.06	646	0.102	**2.49**
XIX	71.4	0.45	0.11	651	0.110	**1.25**
XX	45.8	0.40	0.16	626	0.073	**0.62**
XXI	72.0	0.39	0.20	652	0.110	**0.48**
XXII	80.6	0.37	0.17	661	0.122	**0.54**
Median	71.7	0.33	0.15	652	0.110	**0.50**
**Average**	**80.5**	**0.34**	**0.15**	**661**	**0.120**	**0.66**
Error	29.3	0.09	0.05	29	0.038	**0.53**

SI10. Monte Carlo simulations

The *in vivo* FCS experiments were supported by simulations of Drp1 diffusion in living cells. Also the kinetics of oligomerization was modelled. The simulations were performed using Monte Carlo cell (Mcell) simulator (references 42-44) supported by CellBlender 1.1 environment. Output of the MCell simulations was piped into the FERNET toolkit where FCS timetraces were generated (reference 44). The dimensions of simulation box were set as 5×5×1 μm and the walls of the simulation box were set as reflecting. Figure 4:d (main text) shows the snapshot of the simulation. The focal volume exceeds over the simulation box which imitate the real experiments where the focal volume is limited by the cell's height.

The simulation step was equal to 1 μs for all molecules. In the model two reactions were considered:

$$D + D \leftrightarrow T \qquad \text{(Reaction 1)}$$

and

$$D + D^{*} \leftrightarrow T^{*} \qquad \text{(Reaction 2)}$$

were asterisk denotes the fluorescently tagged molecule. The equilibrium dissociation constant of the both reactions was set equal to K = 0.5μM. The forward rate constant of both reactions was assumed equal to the Smoluchowski equation (SI10.1):

$$k_{on} = 4\pi r_p D N_A \approx 44.2 \times 10^{8} \frac{1}{M\,s}, \qquad \text{(SI10.1)}$$

The backward rate constant was calculated as k_{off} = K k_{on}. For Reaction 1 and Reaction 2 the forward rates used in simulations was given by equation SI10.1. Due to the character of Reaction 1 however the backward rate was set as $k_{off}/2$. This is because the amount of dimer molecules D decreases twice faster than increase of tetramer molecules T. For Reaction 2 the backward rate was equal to k_{off}.

Simulations were run for two cases. Case 1 considered concentrations of fluorescent dimers equivalent to the median of the values obtained experimentally ([D*] = 71.7 (1-p) = 36 nM), where p = 0.5 was a fraction of third component observed in the *in vivo* experiments. In case 2 [D*] = 55.6 (1-0.55) = 25 nM which corresponded to the value obtained for a particular cell (cell X), for comparison of autocorrelation curves.

At each simulation there was an additional fraction of fluorescent molecules that did not take part in the tetramerization reactions but were detectable in the experiments. The concentration of those C molecules was equal to [C] = c_f p, where c_f denotes total concentration of fluorescent molecules (71.7 or 55.6 nM). The equilibrium concentrations of dimers and tetramers were estimated on the basis of initial simulations - the simulation containing only the total amount of unlabelled monomers [M] = 580 nM and labelled [D*] dimers. Number of simulations steps for initial simulations was equal to 5 × 10^4 and corresponded to 50 ms. The equilibrium was obtained after about 1 ms and the equilibrium concentration of the each type of molecule ($[D]_{eq}$, $[D^*]_{eq}$, $[T]_{eq}$, $[T^*]_{eq}$) was calculated as an average concentration for time t > 1 ms. Thus obtained equilibrium concentrations were equal to those calculated from the kinetic equations with approximately 3% of accuracy.

Nest, the real time of simulations were set as 30 s (3×10^7 of iterations). The simulations were run parallelly using 20 different seeds. The output of each Mcell simulation was piped into the Fluorescence Emission Recipes and NumErical routines Toolkit (FERNET) (ref. 45). Such procedure allowed to generate the fluorescence time traces for the point FCS measurement. The focal volume used in FERNET config file had the same dimensions as the focal volume assumed in the *in vivo* experiments. The waist of the focal volume was set as 0.186 μm while its height was equal to 1.11 μm. Brightness of both dimers and tetramers was set as 10^5 counts per molecule per second. The fluorescence time traces were further correlated using the SimFCS software. Fitting of resulting autocorrelation curves was performed using Gnuplot software using the three component model (as was described in SI7).
